# A diagnostic strategy for Parkinsonian syndromes using quantitative indices of DAT SPECT and MIBG scintigraphy: an investigation using the classification and regression tree analysis

**DOI:** 10.1007/s00259-020-05168-0

**Published:** 2021-01-03

**Authors:** Yu Iwabuchi, Masashi Kameyama, Yohji Matsusaka, Hidetoshi Narimatsu, Masahiro Hashimoto, Morinobu Seki, Daisuke Ito, Hajime Tabuchi, Yoshitake Yamada, Masahiro Jinzaki

**Affiliations:** 1 Department of Radiology, Keio University School of Medicine, 35 Shinanomachi, Shinjuku-ku, Tokyo 160-8582 Japan; 2 Department of Diagnostic Radiology, Tokyo Metropolitan Geriatric Hospital and Institute of Gerontology, Tokyo, Japan; 3 Department of Neurology, Keio University School of Medicine, Tokyo, Japan; 4 Department of Neuropsychiatry, Keio University School of Medicine, Tokyo, Japan

**Keywords:** ^123^I-Ioflupane, ^123^I-FP-CIT, CART, Data mining, Artificial intelligence

## Abstract

**Purpose:**

We aimed to evaluate the diagnostic performances of quantitative indices obtained from dopamine transporter (DAT) single-photon emission computed tomography (SPECT) and ^123^I-metaiodobenzylguanidine (MIBG) scintigraphy for Parkinsonian syndromes (PS) using the classification and regression tree (CART) analysis.

**Methods:**

We retrospectively enrolled 216 patients with or without PS, including 80 without PS (NPS) and 136 with PS [90 Parkinson’s disease (PD), 21 dementia with Lewy bodies (DLB), 16 progressive supranuclear palsy (PSP), and 9 multiple system atrophy (MSA). The striatal binding ratio (SBR), putamen-to-caudate ratio (PCR), and asymmetry index (AI) were calculated using DAT SPECT. The heart-to-mediastinum uptake ratio (H/M) based on the early (H/M [Early]) and delayed (H/M [Delay]) images and cardiac washout rate (WR) were calculated from MIBG scintigraphy. The CART analysis was used to establish a diagnostic decision tree model for differentiating PS based on these quantitative indices.

**Results:**

The sensitivity, specificity, positive predictive value, negative predictive value, and accuracy were 87.5, 96.3, 93.3, 92.9, and 93.1 for NPS; 91.1, 78.6, 75.2, 92.5, and 83.8 for PD; 57.1, 95.9, 60.0, 95.4, and 92.1 for DLB; and 50.0, 98.0, 66.7, 96.1, and 94.4 for PSP, respectively. The PCR, WR, H/M (Delay), and SBR indices played important roles in the optimal decision tree model, and their feature importance was 0.61, 0.22, 0.11, and 0.05, respectively.

**Conclusion:**

The quantitative indices showed high diagnostic performances in differentiating NPS, PD, DLB, and PSP, but not MSA. Our findings provide useful guidance on how to apply these quantitative indices in clinical practice.

## Introduction

Atypical parkinsonian syndromes (APS) are characterized by a more rapid progression and poorer prognosis than typical Parkinson’s disease (PD). However, clinicopathologic studies have indicated that patients with APS are underdiagnosed; the clinical diagnostic accuracy is not optimal [1]. For more accurate diagnosis and appropriate treatment of different types of APS, proper utilization of dopamine transporter (DAT) single-photon emission computed tomography (SPECT) and ^123^I-metaiodobenzylguanidine (MIBG) scintigraphy would be valuable.

Quantitative assessments are effective in interpreting DAT SPECT and MIBG scintigraphy, thereby leading to reduced inter-observer disagreement and more accurate diagnoses for Parkinsonian syndromes (PS) [2, 3]. The striatal binding ratio (SBR), putamen-to-caudate ratio (PCR), and asymmetry index (AI) are quantitative indices calculated from DAT SPECT [4–7]. The SBR represents the strength of striatal radioisotope uptake, the PCR represents a change in the shape of striatal uptake, and the AI represents the laterality of striatal uptakes. The heart-to-mediastinum uptake ratios based on the early and delayed images (H/M [Early] and H/M [Delay], respectively) and the cardiac washout rate (WR) are quantitative indices calculated from MIBG scintigraphy [8–12]. The H/M (Early) represents cardiac sympathetic distribution and density and depends on uptake-1 at the sympathetic nerve ending [10, 13]. The WR reflects a release of the initial uptake from the sympathetic nerve endings and is influenced by the re-uptake rate and sympathetic turn-over rate [14]. The H/M (Delay) depends on both the H/M (Early) and WR and comprehensively represents sympathetic function, including the distribution, density, and activity. While these quantitative indices provide useful information for image interpretation, it is necessary to establish the correct usage method by effectively combining these quantitative values.

The classification and regression tree (CART) analysis, which belongs to a family of nonparametric regression methods based on recursive partitioning of data, builds a decision tree structure and classifies subjects to generate groups of patients with similar clinical features [15]. The results of decision tree analysis provide the visualization and interpretation of interactions between factors related to differential diagnosis. However, to the best of our knowledge, no prior clinical studies have assessed or determined a method for using these six quantitative indices in combination as an effective diagnostic strategy for PS.

We hypothesised that proper combined use of these quantitative indices would enable more accurate differential diagnosis. Accordingly, the purpose of this study was to assess the diagnostic impact of each quantitative index using the CART analysis and investigate the proper usage instructions for differentiating patients with PS, including PD, dementia with Lewy bodies (DLB), progressive supranuclear palsy (PSP), and multiple system atrophy (MSA).

## Materials and methods

### Patients

**Fig. 1:**
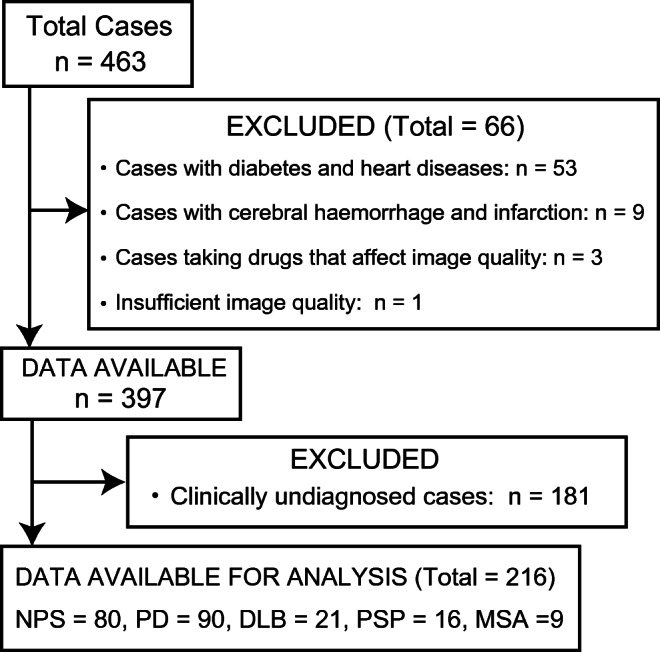
Flow diagram of patient inclusion. Abbreviations: *DLB* dementia with Lewy body, *MSA* multiple system atrophy, *NPS* non-Parkinsonian syndrome, *PD* Parkinson’s disease. *PSP* progressive supranuclear palsy

This single-centre retrospective study included 463 consecutive patients who underwent DAT SPECT and MIBG scintigraphy between December 2013 and April 2019; the interval between the two examinations was less than 1 year. Patients with a disease that would affect DAT and MIBG image quality (e.g., cerebral haemorrhage, brain infarction, diabetes, or heart disease) and those who were taking drugs that would affect the accumulation of DAT and MIBG images (e.g., tricyclic antidepressants, reserpine, labetalol, SSRI, or central nervous system stimulants) were excluded [16, 17]. Patients who were clinically undiagnosed were also excluded, including those who did not meet the diagnostic criteria for any disease or those who were difficult to follow-up due to hospital change. Figure 1 depicts a flow diagram of the study participants (Fig. 1). Patients who were not clinically diagnosed with a degenerative disease and had essential tremor and drug-induced Parkinsonism that improved during follow-up were included in the without PS (NPS) group. Among a total of 463 patients, 97 overlapped with those from our previous study [6].

The institutional review board of the Keio University School of Medicine granted permission for this retrospective review of imaging and clinical data and waived the requirement for obtaining informed consent from the patients (approval number: 20150441).

### Image acquisition and reconstruction

DAT SPECT images were acquired 3 h after injection of ^123^I-Ioflupane (185 MBq) using Discovery NM/CT 670 or Discovery NM 630 (GE Healthcare, Milwaukee, WI) mounted with a FAN beam collimator. Imaging parameters were as follows: matrix size, 128 × 128; pixel size, 4.4 mm; slice thickness, 4.4 mm; and energy window, 159 keV ± 10%. Projection data acquired for 30 min were reconstructed on a Xeleris workstation (GE Healthcare). The ordered-subset expectation maximization method (iterations, 3; subset, 10) and a Butterworth filter (critical frequency, 0.5; power, 10.0) were applied to analyse the SPECT images. Neither attenuation correction nor scatter correction was used.

MIBG planar images of the chest were acquired 15 min and 3 h after injection of ^123^I-metaiodobenzylguanidine (111 MBq) using Discovery NM/CT 670 or Discovery NM 630 (GE Healthcare, Milwaukee, WI) mounted with an extended low-energy general-purpose collimator. The image acquisition time was 5 min, and the imaging parameters were as follows: matrix size, 256 × 256; pixel size, 1.10 mm; zoom, 2; and energy window, 159 keV ± 20%.

### Calculation of quantitative indices of DAT SPECT

**Fig. 2:**
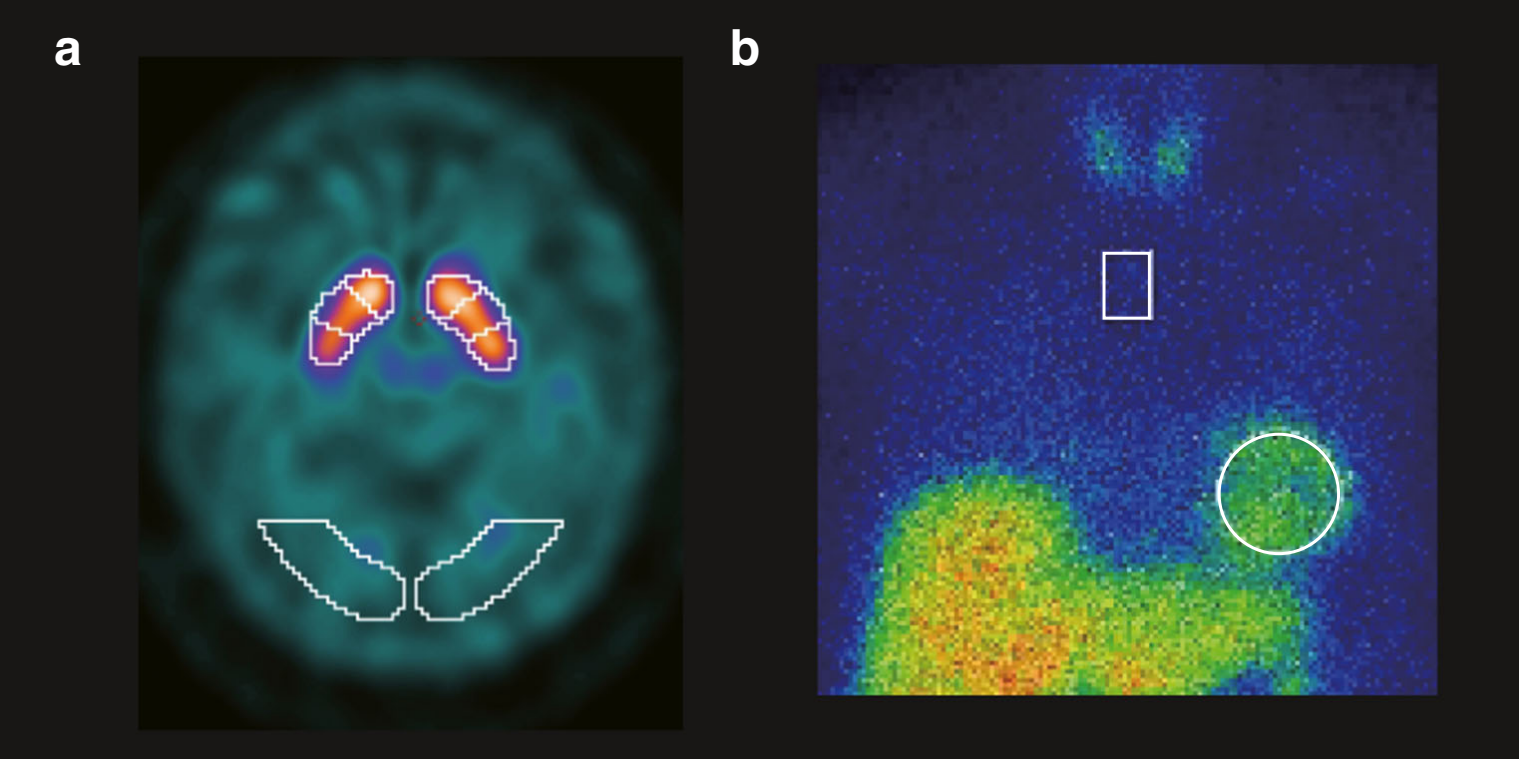
Representative DAT SPECT and MIBG scintigraphy images. **a** A representative VOI setting of DAT SPECT. A commercially available software package DaTQUANT was used. **b** A representative ROI setting of MIBG scintigraphy. A commercially available software package smartMIBG was used. Abbreviations: *DAT* dopamine transporter, *MIBG* ^123^I-metaiodobenzylguanidine, *ROI* region of interest, *SPECT* single-photon emission computed tomography, *VOI* volume of interest

We performed volume of interest (VOI)-based analyses using a commercially available software package: DaTQUANT (GE Healthcare, Little Chalfont, UK) (Fig. 2a). DaTQUANT uses a widespread VOI-based approach that applies a normalized VOI template; two striatal normalized VOIs and two normalized occipital lobe VOIs for the reference background are set automatically. The template is based on the large European multi-centre database of healthy controls for the DAT SPECT (ENC-DAT trial) [18]. This software enables the automatic calculation of the following three types of quantitative indices: the SBR, PCR, and AI [6]. The SBR is defined as mean counts of the striatal VOI (background-subtracted) divided by mean counts of the occipital lobe VOI and represents the count ratio. The PCR is defined as mean counts of the Putamen-VOI divided by mean counts of the Caudate-VOI. The AI is defined as the difference between the SBR of both sides divided by the mean SBR of both sides. Regarding the SBR and PCR indices, the smaller value of both striatums was used for the analysis.

### Calculation of quantitative indices of MIBG scintigraphy

We performed the region of interest (ROI)-based analysis using a commercially available software package: smartMIBG (FUJIFILM Toyama Chemical Co., Ltd., Tokyo, Japan) (Fig. 2b). This software enables the automatic calculation of three types of quantitative indices (H/M [Early], H/M [Delay], and WR) based on the standard method described previously [11, 12]. H/M Early and H/M Delay are defined as the count density of the left ventricular ROI divided by that of the mediastinal ROI on the early and delayed images, respectively. WR is defined as:

$$ \mathrm{WR}=\frac{\left( He- Me\right)-\left( Hd- Md\right)/k}{\left( He- Me\right)} $$

where He and Hd are the counts of the Heart-ROI on the early and delayed images, respectively; Me and Md are the counts of the Mediastinum-ROI on the early and delayed images, respectively; and *k* is a decay coefficient.

### Statistical model

The Kruskal-Wallis test was used to compare the quantitative indices between the NPS and PS (PD, DLB, PSP, and MSA) groups. If there were significant differences, post hoc analysis with Bonferroni correction was performed. The tests were performed using SPSS software (version 25; SPSS Inc., Chicago, IL).

A CART method was used to determine the quantitative indices that could best differentiate the groups of disorders [15]. Regarding the pruning of the decision tree, a complexity parameter was set so as not to exceed the minimum value of *xerror* in cross-validation +1 standard deviation (SD) based on Min-1SE rule. The statistical package R (version 3.2.2; available as a free download from http://www.r-project.org) was used for CART analysis. We used dtreeviz (https://github.com/parrt/dtreeviz, accessed June 23rd, 2020) for better visualization of the decision tree. The feature importance for each quantitative index, known as the Gini importance, was computed based on the optimal decision tree. The sensitivity, specificity, positive predictive value (PPV), negative predictive value (NPV), and accuracy of the quantitative indices were calculated based on the optimal decision tree. Differences with *p* values of < 0.05 (two-sided) were considered statistically significant.

## Results

Among the 463 consecutive patients included, 66 were excluded from the study due to insufficient image quality, and 181 patients who were clinically undiagnosed were also excluded. The remaining 216 patients (median age, 69.4 years; range, 23–91 years; men/women, 114/102), including 136 with PS and 80 without PS (NPS), were included in this study analysis. Among the 136 patients with PS, 90 were diagnosed with PD based on the clinical diagnostic criteria of the UK Parkinson’s Disease Society Brain Bank [19], and the remaining 46 were diagnosed with atypical PS, including clinical DLB (*n* = 21), PSP (*n* = 16), and MSA (*n* = 9), based on the established diagnostic criteria [20–22] (Fig. 1). Table 1 shows the characteristics of the included patients.

**Table 1:** Patient characteristics

Characteristics	Values
Age (years, mean ± SD)	69 ± 11.0
Sex	
Men	114 (52.8%)
Women	102 (47.2%)
Type of disease	
NPS	80 (37.0%)
PD	90 (41.7%)
DLB	21 (9.7%)
PSP	16 (7.4%)
MSA	9 (4.2%)

Abbreviations: *SD* standard deviation, *NPS* non-Parkinsonian syndrome, *PD* Parkinson’s disease, *DLB* dementia with Lewy body, *PSP* progressive supranuclear palsy, *MSA* multiple system atrophy

**Fig. 3:**
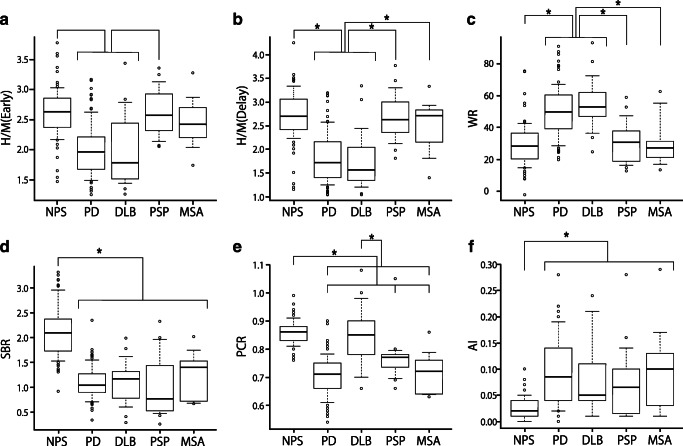
Box-and-whisker plots of the quantitative indices. Asterisks represent significant differences between patients’ groups (*p* < 0.05). Abbreviations: *AI* asymmetry index, *DLB* dementia with Lewy body, *H/M* heart-to-mediastinum activity uptake ratio, *MSA* multiple system atrophy, *NPS* non-Parkinsonian syndrome, *PCR* putamen-to-caudate ratio, *PD* Parkinson’s disease, *PSP* progressive supranuclear palsy, *SBR* striatal binding ratio, *WR* cardiac washout rate

Table 2 shows the mean and SD of six quantitative indices obtained from DAT SPECT and MIBG scintigraphy. Figure 3 shows the box-and-whisker plots of the quantitative indices. The mean H/M (Early) of patients with NPS and PSP were significantly higher than those of patients with PD and DLB (*p* < 0.05; Fig. 3a). The mean H/M (Delay) of patients with NPS, PSP, and MSA were significantly higher than those of patients with PD and DLB (*p* < 0.05; Fig. 3b). The mean WR of patients with NPS, PSP, and MSA was significantly lower than those of patients with PD and DLB (*p* < 0.05; Fig. 3c). The mean SBR of patients with NPS was significantly higher than that of patients with PD, DLB, PSP, and MSA (p < 0.05; Fig. 3d). The mean PCR of patients with NPS was significantly higher than that of patients with PD, PSP, and MSA (p < 0.05; Fig. 3e), and the mean PCR of patients with DLB was significantly higher than that of patients with PD and MSA (p < 0.05; Fig. 3e). The mean AI of patients with NPS was significantly lower than that of patients with PD, DLB, PSP, and MSA (p < 0.05; Fig. 3f).

**Table 2:** Mean and standard deviations of quantitative indices in the five clinical groups

	NPS	PD	DLB	PSP	MSA
H/M (Early)	2.61 ± 0.43	1.99 ± 0.44	1.98 ± 0.58	2.61 ± 0.40	2.46 ± 0.44
H/M (Delay)	2.72 ± 0.54	1.82 ± 0.54	1.73 ± 0.62	2.67 ± 0.52	2.50 ± 0.58
WR	29.3 ± 13.5	50.1 ± 16.1	55.1 ± 16.0	31.0 ± 13.3	31.1 ± 16.4
SBR	2.13 ± 0.51	1.08 ± 0.33	1.10 ± 0.43	1.01 ± 0.63	1.24 ± 0.49
PCR	0.85 ± 0.10	0.71 ± 0.07	0.84 ± 0.11	0.77 ± 0.08	0.72 ± 0.08
AI	0.03 ± 0.02	0.09 ± 0.06	0.08 ± 0.07	0.08 ± 0.07	0.10 ± 0.09

Abbreviations: *NPS* non-Parkinsonian syndrome, *NPV* negative predictive value, *PD* Parkinson’s disease, *DLB* dementia with Lewy body, *PSP* progressive supranuclear palsy, *MSA* multiple system atrophy, *H/M* heart-to-mediastinum activity uptake ratio, *WR* cardiac washout rate, *SBR* striatal binding ratio, *PCR* putamen-to-caudate ratio, *AI* asymmetry index

**Fig. 4:**
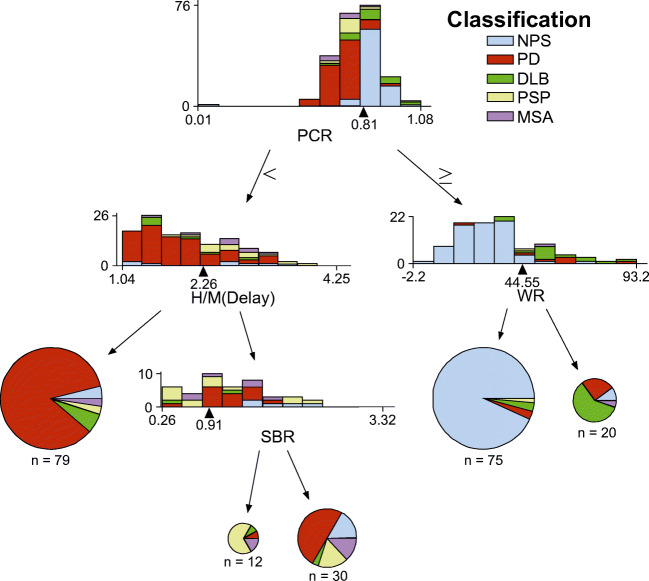
Optimal decision tree used to classify the patient groups. The bar graphs show the distribution of the patient groups at the optimal quantitative index in each node. The arrowheads in the bar graphs represent the cutoff values for each quantitative index. The pie charts represent the proportion of patients’ groups in terminal nodes. Abbreviations: *AI* asymmetry index, *DLB* dementia with Lewy body, *H/M*, heart-to-mediastinum activity uptake ratio, *MSA* multiple system atrophy, *NPS* non-Parkinsonian syndrome, *PCR* putamen-to-caudate ratio, *PD* Parkinson’s disease, *PSP* progressive supranuclear palsy, *SBR* striatal binding ratio, *WR* cardiac washout rate

Figure 4 shows the results of the optimal decision tree using the CART analysis. Among the six quantitative indices, the PCR, WR, H/M (Delay), and SBR indices were used for the optimal decision tree, whereas the H/M (Early) and AI indices were not incorporated. The feature importance of the PCR, WR, H/M (Delay), and SBR indices were 0.61, 0.22, 0.11, and 0.05, respectively. The cutoff values were 0.81 for the PCR index, 44.55 for the WR index, 2.26 for the H/M (Delay) index, and 0.91 for the SBR index. The obtained optimal decision tree model demonstrated that the NPS, PS, DLB, and PSP groups could be differentiated using the quantitative indices, but the MSA group could not. Table 3 provides a summary of the sensitivity, specificity, PPV, NPV, and accuracy using the optimal decision tree.

**Table 3:** Sensitivity, specificity, PPV, NPV, and accuracy of the four groups based on a decision tree

	Sensitivity (%)	Specificity (%)	PPV (%)	NPV (%)	Accuracy (%)
NPS	87.5 (70/80)	96.3 (131/136)	93.3 (70/75)	92.9 (131/141)	93.1 (201/216)
PD	91.1 (82/90)	78.6 (99/126)	75.2 (82/109)	92.5 (99/107)	83.8 (181/216)
DLB	57.1 (12/21)	95.9 (187/195)	60.0 (12/20)	95.4 (187/196)	92.1 (199/216)
PSP	50.0 (8/16)	98.0 (196/200)	66.7 (8/12)	96.1 (196/204)	94.4 (204/216)

Abbreviations: *PPV* positive predictive value, *NPV* negative predictive value, *NPS* non-Parkinsonian syndrome, *PD* Parkinson’s disease, *DLB* dementia with Lewy body, *PSP* progressive supranuclear palsy

**Fig. 5:**
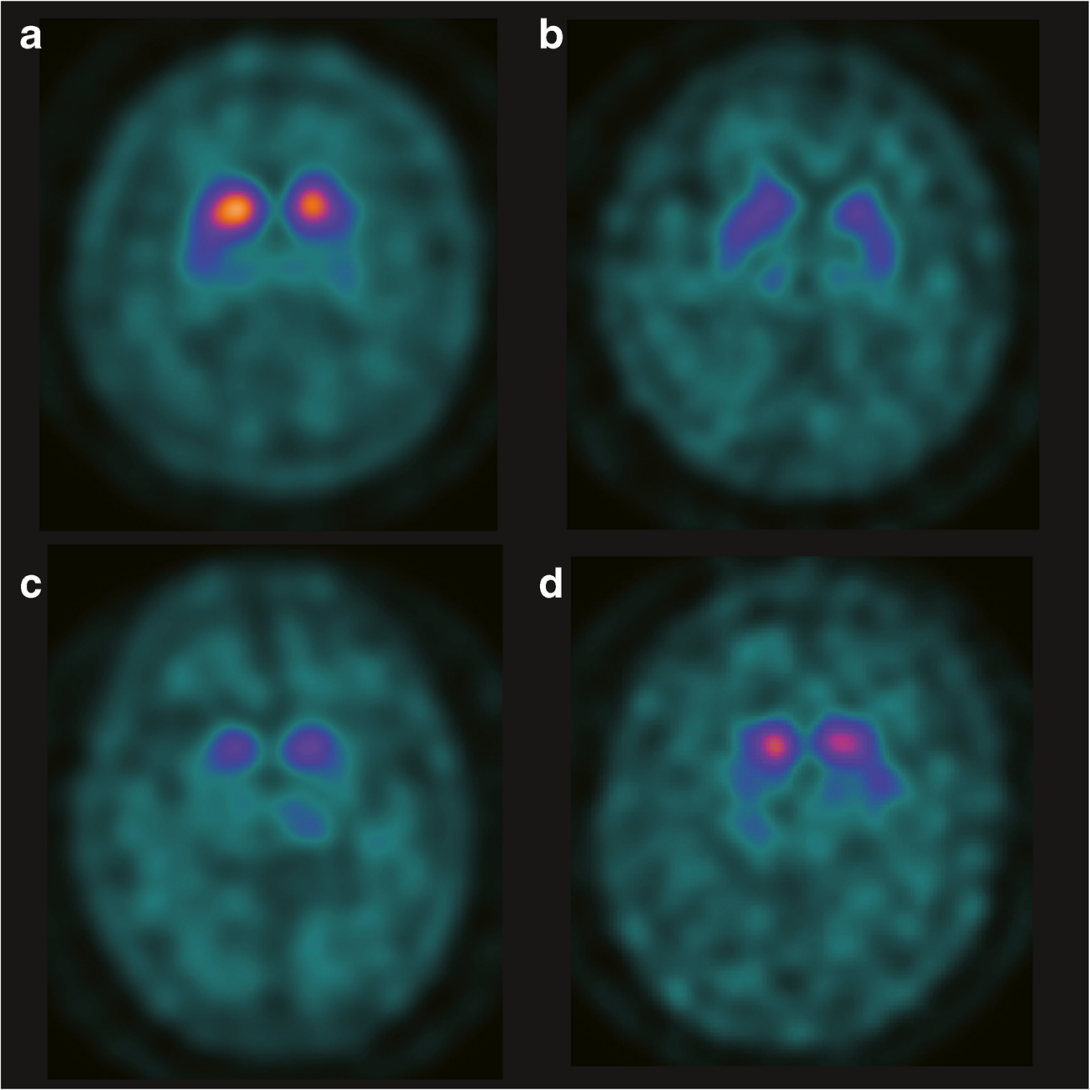
DAT SPECT images of representative cases of PD, DLB, PSP, and MSA. **a** A representative case of PD (aged 64 years, Female). **b** A representative case of DLB (aged 79 years, Female). **c** A representative case of PSP (aged 86 years, Female). **d** A representative case of MSA (aged 56 years, Male). Abbreviations: *DLB* dementia with Lewy body, *MSA* multiple system atrophy, *PD* Parkinson’s disease, *PSP* progressive supranuclear palsy

Figure 5 shows the representative DAT SPECT images of patients with PD, DLB, PSP, and MSA. In the case of PD, posterior striatum uptake decreased (SBR = 1.65, PCR = 0.60, AI = 0.14, respectively, Fig. 5a). In the case of DLB, striatum uptake decreased diffusely, including within the caudate nucleus (SBR = 0.84, PCR = 0.90, AI = 0.11, respectively, Fig. 5b). In the case of PSP, a marked decrease in striatal uptake was observed (SBR = 0.53, PCR = 0.69, AI = 0.09, respectively, Fig. 5c). In the case of MSA, no disease-specific findings were present (SBR = 1.40, PCR = 0.64, AI = 0.06, respectively, Fig. 5d).

## Discussion

This study investigated the diagnostic performances of quantitative indices obtained from DAT SPECT and MIBG scintigraphy for PS using the CART analysis. The findings demonstrated that patients with NPS, PD, DLB, and PSP could be differentiated using the optimal decision tree model with encouraging diagnostic performances.

In patients with NPS, the shape of the striatal uptake was maintained as a normal comma-like shape, and the myocardial sympathetic activity was normal; the PCR and WR could be used to interpret these findings and differentiate patients with NPS. In patients with PD, the uptake of the posterior striatum deteriorated, and the myocardial sympathetic function was abnormal; the PCR and H/M (Delay) indices could be used to interpret these findings. Although the AI index was expected to be useful in differentiating patients with PD [4, 23], our analysis did not show that the AI index played a significant role in diagnosing patients with PD, probably because the left-right differences became less noticeable as the symptoms progressed in some PD patients or those with other PS who also had left-right differences to a certain extent (Fig. 3f). In addition, the PCR and WR indices could be used to differentiate patients with DLB. Our results demonstrated that the PCR index of patients with DLB tended to be higher than that of patients with PD (Figs. 3e, 5a, and b), indicating that the uptake of the anterior striatum decreases in patients with DLB compared with those with PD. We speculate that patients with PD, in whom the symptoms mainly occur due to motor dysfunction, have decreased uptake in the striatum posteriorly, whereas those with DLB, in whom the cognitive function is often impaired, have decreased uptake in the caudate nucleus. Consistently, previous studies showed that the caudate was coactive with a higher level of the cognitive areas, whereas the putamen showed a high degree of coactivation with primary cortical motor areas [24–26]. Although Klein et al. reported no significant differences in 18-fluorodopa PET findings between PD and DLB patients [27], they did not use the PCR index in their assessment. Our results showed that the quantitative indices of MIBG scintigraphy did not differ significantly between PD and DLB patients. Consistently, Uchiyama et al. also found no major difference in MIBG uptake between PD and DLB patients [10]. Moreover, in patients with PSP, the myocardial sympathetic nerve function was maintained based on the findings of MIBG scintigraphy, and the DAT function was deteriorated based on the findings of DAT SPECT; the PCR, H/M (Delay), and SBR indices were key indices for interpreting these findings. In particular, a low SBR value would suggest the possible diagnosis of PSP (Fig. 5c), and the findings are consistent with those in previous studies [28, 29]. These findings suggest that it is necessary to perform both DAT SPECT and MIBG scintigraphy in clinical practice to obtain a reliable clinical diagnosis for PS.

Our results showed that among the six quantitative indices, the PCR index had the most important role in the optimal decision tree, and the H/M (Delay) and WR index also played important roles in distinguishing patients with PD and DLB. The PCR index has been suggested to be particularly valuable as it is background-, age-, and camera-independent [3]. Furthermore, our result demonstrated that the PCR index played a major role in differentiating not only the NPS group but also the DLB group from other groups (Fig. 3e and Fig. 4). Matesan et al. reported that the PCR index might not be a reliable numeric marker in the interpretation of DAT SPECT [5], but their study did not include patients with NPS and DLB; this factor may explain the difference between our findings and theirs. Our result also showed that the H/M (Delay) and WR indices played more important roles than the H/M (Early) index in the optimal decision tree. Kashihara et al. previously found that a reduced H/M ratio was more marked on the delayed image than on the early image in patients with PD or DLB [9], suggesting that both patients with PD and those with DLB not only have decreased cardiac sympathetic nerve terminals but also enhanced spillover of, or reduced ability to preserve MIBG or norepinephrine in their cardiac sympathetic nerve terminals; this finding indicates that dysfunction of the cardiac sympathetic nerve terminals may precede their loss. Our results also support this hypothesis.

To our knowledge, this study is the first to use the CART analysis for comparing the six types of indices from DAT SPECT and MIBG scintigraphy. Although several studies have evaluated the utility of the combined use of DAT SPECT and MIBG scintigraphy [23, 29–33], no study has applied the six quantitative indices in differentiating patients with NPS, PD, DLB, PSP, and MSA. Some patients included in this study overlapped with those included in our previous study [6]. In the previous study, we assessed the utility of the support vector machine to combine the quantitative indices of DAT SPECT for differential diagnosis between NPS and PS; however, we did not use MIBG scintigraphy in the assessment, and the differentiation among PD, DLB, PSP, and MSA was not determined.

This study has several limitations. First, the diagnoses of PS and NPS were clinical diagnoses and were not pathologically evidenced. Second, this study did not stratify patients according to the severity of disease. This may have affected the diagnostic performance of each quantitative index. Third, we excluded 66 cases because we could not guarantee the image quality. While this is a relatively large number, functional images are easily modified by background diseases and drugs. Therefore, we decided to exclude them to prioritize ensuring image quality. Fourth, this was a single-centre study, and institution-specific factors might limit the generalisability of our results. Thus, a multi-centre study with a larger number of participants is warranted to evaluate the efficacy of the decision tree for accurate diagnoses.

## Conclusion

Using the diagnostic strategy based on the optimal decision tree model, quantitative indices from DAT SPECT and MIBG scintigraphy showed high diagnostic performances in differentiating among NPS, PS, DLB, and PSP, but not MSA. The diagnostic strategy would provide useful guidance on how to use these quantitative indices in clinical practice.

## Data Availability

All data generated or analysed during this study are included in this published article.
